# Using the World Health Organization’s Disability Assessment Schedule (2) to assess disability in community-dwelling stroke patients

**DOI:** 10.4102/sajp.v73i1.343

**Published:** 2017-05-19

**Authors:** Ayorinde I. Arowoiya, Toughieda Elloker, Farahana Karachi, Nondwe Mlenzana, Lee-Ann Jacobs-Nzuzi Khuabi, Anthea Rhoda

**Affiliations:** 1 Department of Physiotherapy, Faculty of Community & Health Science, University of the Western Cape, South Africa; 2 Division of Occupational Therapy, Department of Interdisciplinary Health Sciences, University of Stellenbosch, South Africa

## Abstract

**Background:**

Measurement of the extent of disability post-stroke is important to determine the impact of disability on these individuals and the effectiveness of interventions aimed at reducing the impact of their disability. Instruments used to measure disability should, however, be culturally sensitive.

**Objective:**

The aim of this study was to conduct a disability assessment using the World Health Organization’s Disability Assessment Schedule 2.0 (WHODAS).

**Methods:**

A cross-sectional design was used. The study population included a conveniently selected 226 stroke patients living within community settings. These patients were followed up 6–12 months following the onset of the stroke and are currently residing in the community. Disability was measured using the WHODAS 2.0 and the data were analysed using descriptive and inferential statistics in Statistical Package for Social Sciences (SPSS). The WHODAS 2.0 enabled the assessment of disability within the domains of cognition, mobility, self-care, getting along with others, household activities, work activities and participation. Ethical clearance for the study was obtained from the University of the Western Cape.

**Results:**

In this sample, the domain mostly affected were household activities, with 38% having extreme difficulty with conducting these activities. This was followed by mobility (27%) and self-care (25%) being the domains that participants also had extreme difficulty with. Getting along with others was the domain that most (51%) of the participants had no difficulty with. ANOVA one-way test showed no significant association of participation restrictions with demographics factors.

**Conclusion:**

Rehabilitation of patients with stroke should focus on the patient’s ability to engage in household activities, mobility and self-care.

## Introduction

Stroke has been identified as a leading cause of mortality and morbidity (Zhang et al. 2011). A stroke is a sudden and often traumatic major life event that usually occurs with minimal warning and, for many, results in life-changing consequences with which affected people must cope (Anonymous 2005). In sub-Saharan Africa, stroke is considered to be the most prominent type of vascular disease (Lozano, Naghavi and Foreman 2012). According to the American Heart Association (2011), the effect of a stroke is devastating, not only for the survivor but also for the primary caregiver and family who are often subjected to severe stress. Many patients survive the initial event but are left with disability and face the consequent challenge of reintegrating into residential and community living (Go et al. 2014). The World Health Organization’s conceptual framework of disability, known as the International Classification of Functioning, Disability and Health (ICF), views disability as a multi-dimensional concept relating to the health condition, body functions and structures, activity limitations, participation restrictions and contextual factors which include environmental and personal factors (Wasserman, De Villiers & Bryer 2009). In relation to a stroke, common impairments identified in individuals with stroke include movement, speech and cognitive impairments (Bryer, Villiers & Wasserman 2009), while activity limitations include challenges with walking and conducting activities of daily living (Bryer, Tipping & De Villiers 2006). With regard to participation, patients post-stroke lack the ability to return to work and engage in social activities, while environmental barriers that have been identified include physical and attitudinal environmental challenges (Herman 2006). It is, however, important not only to conceptualise disability in terms of conditions such as a stroke, but also be able to measure the extent thereof, and to generate information about the individuals’ health needs and the effectiveness of interventions aimed at reducing disability, and thus improving the health of persons thereby affected (Ustun et al. 2010).

Various national and international guidelines have been published with regard to the assessment and management of disability, focusing not only on the acute phase of the disease, but also on the broader context of disability (Geyh et al. 2004). To assess disability post-stroke at the level of various domains, the ICF authors have mainly used standardised outcome measures which often only measure one aspect of the ICF (Ustun et al. 2010). Ustun et al. (2010) highlighted that it is however important for standardised measures used to assess disability, within the framework of the ICF, to ‘be linked conceptually and operationally to the ICF to allow comparisons across different cultures and populations’.

A number of studies have been conducted to measure and explore disability in stroke patients living in African countries including Rwanda, Tanzania and South Africa (Sousa, Ferri & Acousta, 2009; Rhoda et al. 2015). The studies conducted were qualitative as well as quantitative. The quantitative studies, based within the framework of the ICF, used a number of standardised outcome measures, such as the Barthel Index and Stroke Specific Impact Scale, which were not specifically designed with the ICF in mind. In addition, the study conducted by Rhoda et al. (2015) qualitatively explored participation restrictions experienced by stroke patients. These studies could, therefore, have missed some of the constructs included in the ICF in the measurement of disability post-stroke. This study, therefore, aims to measure disability post-stroke as defined in the ICF using the World Health Organization’s Disability Assessment Schedule 2.0 (WHODAS 2.0). The instrument referred to in this study is WHODAS 2.0 and is based on a comprehensive theoretical concept of human functioning and disability, incorporating social and environmental aspects of disability which constitute key components of disability assessment (Kulnik & Nikoletou 2014).

## Methodology

### Setting

The study was conducted at Community Health Centres (CHCs) in the Metro District Health Service of the Western Cape. These centres are responsible for providing primary health care in the region. The majority of the population attend these centres for medical management (Irlam, Levin & Reagan 2003). These services are predominantly utilised by individuals from disadvantaged communities, low socio-economic class, poor knowledge of health and low levels of education [Southern Africa Stroke Prevention Initiative (SASPI) 2004]. A total of 13 CHCs were used to obtain data for this study, which were selected based on availability (convenience) of the CHCs. This sampling method was adopted because of availability of the participants during the course of the study (Babbie 2001). The use of convenience sampling, (i.e. a type of nonprobability sampling) has the limitation of selection bias. Selection bias limits the generalisations and inferences that could be made about the entire population as the entire population was not represented by the sample (Terre Blanche, Durrheim & Painter 2006)

### Population and sampling

All stroke participants attending the CHCs and residing in a community setting for at least 6–12 months were invited to participate in the study. Participants were therefore sampled by means of convenience. Participants were excluded from the study if they suffered from severe cognitive deficits and speech impairment, which was determined at the first screening stage of the interview. Participants’ medical records were checked for any diagnosis of aphasia and confirmed by the physiotherapist at the CHC. A total of 226 stroke participants comprised the sample for this study.

### Study design

A cross-sectional quantitative design was used. It was cross-sectional in nature, because (1) the study viewed the functional status of a given population, in a specific location and at a certain point in time and (2) information from the participants was collected without manipulating the study environment, which was the stroke patient living within the community (Kohlmann & Schmidt 2008).

### Instrumentation

The WHODAS 2.0 descriptive quantitative interviewer administered questionnaire was used in this study. The WHODAS 2.0 was developed by the World Health Organization and is used widely as an assessment tool for disability. The instrument assesses disability within six domains of functioning, namely: cognition, mobility, self-care, interpersonal relationships, life activities and participation in society. This instrument consists of a demographic component, followed by 36 questions where participants are required to think of the past 30 days only when attempting to answer. Participants are asked how much difficulty they have with any given task, and requested to rate each item based on the 5-point Likert scale (1–5), where 1 indicates no difficulty and 5 indicates extreme difficulty. This is used to determine participants’ functional ability (Ustun 2010). The WHODAS 2.0 has been found to be both reliable (intra-class correlation coefficient: 0.98) and valid (Cronbach’s alpha, α 0.86) with high internal consistency.

### Procedure

Data collection commenced once ethics approval and permission to conduct the study was obtained from all the necessary authorities. The freedom to withdraw, confidentiality of information and anonymity were clearly explained, after which the participants were asked to sign an informed consent form. Ethical consideration granted by the University of the Western Cape, with registration no: 13/6/32. The rehabilitation professionals from the CHCs of interest assisted with recruiting suitable participants for the study, for which the inclusion criteria were used in the recruitment process. Participants receiving treatment at the CHCs were contacted and invited to partake in the study. Data collection commenced at the CHCs’ or participants’ residence, as preferred by the participants themselves. All research assistants underwent training, guided by the WHODAS 2.0 User Manual prior to data collection. Data were collected over a period from May 2013 to July 2015. The interviewer administered 36 items and WHODAS 2.0 was used in collecting the data, which was conducted in face-to-face interviews with the participants. All questionnaires were available in English, Afrikaans and isiXhosa, and researchers were available to conduct the interviews in the participants’ preferred language.

### Analysis

The Statistical Package for Social Sciences (SPSS) Version 21.0 was the software used for descriptive and inferential data analysis. An ANOVA one-way test was used to determine the association between the demographics statistics and participation restrictions. Alpha level was set at 0.05.

## Results

### Demographics of the study population

The demographic profile of the study participants comprises 52.2% males (*n* = 118), with the most common age group affected by stroke in this sample being 60–69 years, as indicated by 30.5% of participants (*n* = 69). With regard to education, 47.3% of participants (*n* = 107) are secondary school certificate holders, and currently 45.6% (*n* = 103) are unemployed as a result of their stroke. The largest proportions of participants (46%) are currently married.

### The extent of general difficulties for the stroke patients

Table 1 is a representation of the extent of difficulty as experienced by the majority of participants, in each domain of the WHODAS. The participants’ disabilities in this study are tabulated below.

**TABLE 1:** The extent of functional limitation for the patients.

Domain of participation	Level of difficulty	Percentage (*n* = 226)
Cognition		
Concentration	Mild to moderate	34.1 (77)
Remembering important things	Mild to moderate	39.8 (90)
Problem solving	Mild to moderate	34.9 (79)
Learning new tasks	Mild to moderate	31.4 (71)
General understanding	Mild to moderate	25.7 (58)
Conservations	Mild to moderate	28.3 (64)
Mobility		
Standing for long periods	Severe to extreme	55.8 (125)
Standing up from sitting	Mild to moderate	47.3 (106)
Moving around at home	Mild to moderate	39.8 (90)
Getting out of the home	Mild to moderate	37.2 (88)
Walking a long distance	Severe to extreme	64.5 (145)
Self-care		
Washing the whole body	Severe to extreme	40.8 (91)
Getting dressed	Mild to moderate	27.2 (61)
Eating	Mild to moderate	24.3 (55)
Staying by yourself	Severe to extreme	50.9 (113)
Getting along with people		
Dealing with unknown people	Mild to moderate	25.7 (58)
Maintaining a friendship	Mild to moderate	22.1 (50)
Getting along with closer people	Mild to moderate	16.4 (37)
Making new friends	Mild to moderate	28.8 (65)
Sexual activities	Severe to extreme	44.1 (86)
Household activities		
Taking care of household responsibilities	Severe to extreme	40.2 (85)
Performing most important household tasks well	Severe to extreme	44.3 (93)
Getting all household work done	Severe to extreme	46.7 (98)
Getting household work done quickly	Severe to extreme	57.2 (120)
Work or school activities		
Day-to-day work	Severe to extreme	50.0 (3)
Doing important work tasks well	Mild to moderate	50.0 (3)
Getting all work done that needed to be done	Severe to extreme	50.0 (3)
Getting work done as soon as possible	No difficulty	60.0 (3)
Participation		
Joining in community activities	Severe to extreme	34.5 (78)
Barriers in the community	Severe to extreme	39.4 (89)
Living with dignity	No difficulty	44.2 (100)
Time consumption on health and its consequence	Mild to moderate	52.6 (119)
Emotional fluctuations because of health condition	Severe to extreme	53.5 (121)
Financial implications	Severe to extreme	48.2 (109)
Burden on family	Severe to extreme	37.2 (84)
Engaging in relaxation/leisure activities	Severe to extreme	39.4 (89)

*Source*: WHO, 2001

In the domains of cognition and getting along with people, the largest percentages of participants reported no difficulty with activities (not presented in the table above). However, smaller percentages reported mild to moderate difficulties with tasks involving concentration (34.1%), remembering important things (39.8%), problem solving (34.9%), interacting with friends (16.4%) and unknown people (25.7%), as well as maintaining relationships (22.1%). It is noted that the majority of participants (44.1%) reported severe to extreme difficulties with sexual activities.

In the domain of mobility, the majority of participants reported severe to extreme difficulties with standing for long periods (55.8%) and walking a long distance (64.5%), such as a kilometre, 12 months post-stroke. Participants were restricted in self-care activities such as staying on their own for a few days (50.9%) and washing their entire body (40.8%), as they found this to be extremely difficult. The largest proportion of participants, a total of 120 (57.2%), were reported to have severe to extreme difficulties with household tasks (40.2%) and aspects related to these activities.

The majority of participants were not involved in any work or school activities because of their health condition and indicated problems with participation in life situations, such as joining in community activities (34.5%), barriers affecting community participation (39.4%), emotional instability (53.5%), financial strain (48.2%), increased burden on family (37.2%), and leisure and relaxation dysfunction (39.4%).

**FIGURE 1:**
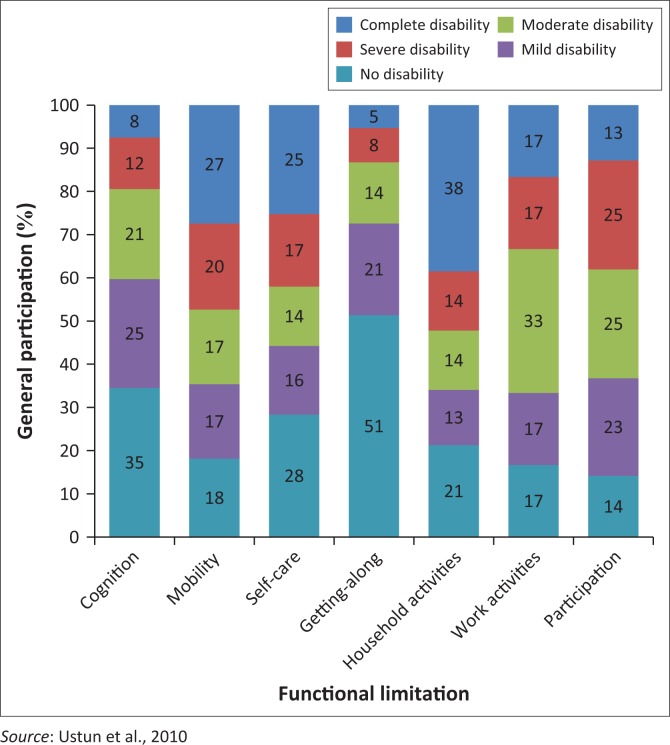
Extent of the patients’ functional limitation.

The extent of the patients’ general participation has been outlined in Figure 1, where 0 indicates no disability and 100 indicates full disability (Ustun et al. 2010).

Figure 1 shows evidence that the general population is least affected in the domains of getting along with people and cognition, in which they display minimal disability. The domains of participation and work activities were more challenging for individuals, as indicated by moderate disability, while the domains requiring individuals to engage physically, that is, household activities, mobility, participation and self-care, were indicated as severe to complete disability for the study sample.

### Association between demographic factors and participation restrictions

An ANOVA was conducted to check for association between the variables, independent variables (e.g. age, gender, education level, marital status and main work status) and the dependent variable (e.g. participants living in the community). The alpha level was set at 0.05; none was recorded to be significant. The results are summarised in Table 2.

**TABLE 2:** Association between demographics factors and participation.

Variable	*F*	*P*
Age	0.75	0.880
Gender	1.92	0.167
Years of education	2.09	0.082
Marital status	0.66	0.653
Main work status	1.68	0.125

*Source*: ANOVA one-way test

## Discussion

The aim of this study was to present information relating to the disability of stroke patients at 6–12 months post-stroke, living within a community in the Western Cape, South Africa, using the WHODAS 2.0. The study revealed the disability experienced by these stroke patients in the domains of cognition, mobility, self-care, life activities and participation. These restrictions are discussed in more detail below.

Participants in this study experienced mild to moderate cognitive impairments, especially in the domain of remembering an important thing, which has been found to have a negative impact on their quality of life (QOL); these impairments will impede heavily on their social participation, leisure activities, employment, independence, daily activities, mood and identity in the community (Pinquart & Sorensen 2006).

All participants demonstrated difficulties with mobility, including walking a long distance such as a kilometre, which was extremely difficult for the majority of participants. According to Lord et al. (2008), who also reported a loss in independence following a stroke, the attainment of independent community ambulation was a challenging rehabilitation goal. This is because, if patients do not have adequate ambulatory ability, this directly affects their ability to participate in the community (Taylor et al. 2006). Walking is an important human activity which enables us to be productive and participative members of a community (Ada et al. 2009). Therefore, difficulty in mobility of these patients will impede on their ability to participate in society in general.

The results showed that the majority of participants reported severe difficulty with staying on their own (50.9%) and washing their body (40.9%) post-stroke, while more than half encountered absolutely no problems with eating by themselves. According to the study by Mayo et al. (2002), 54% of participants reported difficulty with higher-level functional activities of daily living such as dressing and bathing. Stroke limits activities of daily living thereby reducing participation in the community.

From the results shown, 44.1% of participants had severe problems with sexual activities. According to Korpelainin et al. (1998), the study also reported a low quality of sexual life and a marked decline in sexuality after a stroke. The problems that were identified in the study include a decline in libido and coital frequency in both genders, a decline in vaginal lubrication and orgasmic ability in women, and poor or absent erection and ejaculation in men. This implies that dissatisfaction and emotional decline will be reported among participants who will find it difficult to resume their normal active sexual life, which could result in break-ups in relationships because of their inability to resume their usual sexual activities.

Participants experienced severe to extreme difficulties with household responsibilities. Moreover, a below average number of participants reduced or missed their housework completely for 26–30 days in a month, while an above average number of participants completely missed their housework for 5 days in a month. This implies that participants are not able to engage in household activities as they did before, and this could impose more stress on the family members and the need to employ a house helper or caregiver which could result in further financial implications.

Only three participants in this study were employed, and all reported great difficulty with their day-to-day work. Living with a disability can interfere with a person’s ability to participate actively in economic and social life (Phillips & Noumbissi 2004). The inability to return to work post-stroke has been reported in various studies (Varona et al. 2004; Vestling, Tufvesson & Iwarsson 2003; Rhoda, Mpofu & DeWeerdt 2009) and, as a result, might put a strain on the stroke patient’s finances. Considering their disability status, which necessitates more finances in taking care of their health, these participants would likely be in a financial crisis and unable to cater for their needs.

Joining in community activities was severe to extremely difficult for participants (34.5%). This could be as a result of severe barriers in the community affecting participation, as reported by 39.4% of the sample. Hammel et al. (2006) elaborated that barriers include inaccessible entryways, bathroom and transportation systems, door thresholds and lack of handrails affecting community participation for stroke survivors. The inability to participate in community activities could have an adverse effect on their relative QOL. This will cause stroke patients to stay indoors more, encouraging social isolation which could lead to depression. Studies have found that the ability to return to an acceptable lifestyle, participating in both social and domestic activities, is important for patient satisfaction and perceived QOL post-stroke (Bendz 2003; Clark & Smith 1999; Jaracz & Kozubski 2003; Kim & Johnston 2011).

The study showed that 53.5% of participants were severely affected emotionally. Concurrently, a study conducted by Goodman, Schlossberg and Anderson (2006) was of the opinion that emotional distress is a crucial factor impacting on participation. Because of the inability to perform certain activities and fulfil roles post-stroke, these duties are often taken over by family or friends. Consequently, role changes occur as these activities are now being completed by someone else (Maleka, Stewart & Hale 2012). This can result in an altered emotional state and eventually depression (Dowswell et al. 2000).

A large proportion (48.2%) encountered severe financial strain because of their health condition. This is consistent with findings from a literature review by Radford and Walker (2008), which stated that people who cannot return to employment face a lifetime of dependence on their families and the state. This might place a burden on the families of these survivors and increases their financial strain.

The participants’ family (37.2%) experienced extreme difficulty coping with the participants because of their health condition, and an increased burden on the family was also reported especially by relatives, younger children and spouses. According to Lynch et al. (2008), who stated the importance of social relationships, this was reported to be critical in the survival of patients after a stroke and is of critical importance to their QOL. This is indicative that stroke patients, who report difficulty in coping by themselves, require social support and understanding of family members to encourage better recovery and participation in society.

A total of 39.4% of participants reported severe difficulty doing things by themselves for relaxation, which was in line with a quantitative study which revealed that distress among participants was associated with the loss of hobbies that had previously been a source of pleasure and achievement (Ch’Ng, French & Mclean 2008). This implies that the participants face great challenges with recovery and with disability after a stroke, and that the ability to engage in things that give them pleasure or aid in relaxation, is decreased.

## Conclusion

The study revealed that stroke patients have limited functional restoration, even after several months post-stroke, and this impeded heavily on functional, environmental, societal and leisure activities. The increased burden on family, the emotional stress, the feeling of low self-esteem, increased depressive mood, reduced social gathering because of aphasia, loss of memory, mobility difficulty, self-care difficulty, maintaining friendship, low libido in sexual activities, reduced household activities and increased financial strain, had profound effects on functional restoration and participation in society. Therefore, the disability and health assessment of stroke patients living in the community are greatly affected even after several months of the stroke having taken place.

## Limitations of the study

The limitations of the study are as follows:

- Because of the fact that some of the participants had had their stroke over 10 years ago, some could not remember all the information. The participants might, therefore, have stated only what they could remember and some valuable information may have been missed as a result.
- The missing data were managed by means of a summation of the domain, if only one set of data was missed, but in a case of two sets of data being missed, the missing data were managed by case deletion.
- In situations whereby the participants involved were aphasic, or if he or she could not remember things, the patients’ relatives were interviewed; therefore, some valuable information might not have been given accurately.
- Convenience sampling was utilised and this can influence selection bias. As a result, the outcome of this study cannot be generalised to the entire stroke population. This study design was only able to measure stroke patients at a specific point in time without manipulating the environment.
- In future, cohort studies follow-up need to be conducted to determine the functioning of the stroke patient at one year post-stroke, and also to identify changes that occur over time. Randomised controlled trials are recommended to determine distinctive relationships between variables post-stroke.
